# Characterization of *Bactrocera dorsalis* Serine Proteases and Evidence for Their Indirect Role in Insecticide Tolerance

**DOI:** 10.3390/ijms15023272

**Published:** 2014-02-21

**Authors:** Ming-Zhe Hou, Guang-Mao Shen, Dong Wei, Ya-Li Li, Wei Dou, Jin-Jun Wang

**Affiliations:** Key Laboratory of Entomology and Pest Control Engineering, College of Plant Protection, Southwest University, Chongqing 400716, China; E-Mails: houzi3060520@126.com (M.-Z.H.); blackaet@163.com (G.-M.S.); dong_wei1988@yahoo.com (D.W.); Houzi15213344970@gmail.com (Y.-L.L.); anwdou@gmail.com (W.D.)

**Keywords:** *Bactrocera dorsalis*, serine protease, β-Cypermethrin, protease activity, expression profiles

## Abstract

The oriental fruit fly *Bactrocera dorsalis* (Hendel) causes devastating losses to agricultural crops world-wide and is considered to be an economically important pest. Little is known about the digestive enzymes such as serine proteases (SPs) in *B. dorsalis*, which are important both for energy supply and mitigation of fitness cost associated with insecticide tolerance. In this study, we identified five SP genes in the midgut of *B. dorsalis*, and the alignments of their deduced amino acid sequences revealed the presence of motifs conserved in the SP superfamily. Phylogenetic analyses with known SPs from other insect species suggested that three of them were trypsin-like proteases. Analyses of the expression profiles among the different developmental stages showed that all five genes were most abundant in larvae than in other stages. When larvae were continuously fed on diet containing 0.33 μg/g β-Cypermethrin, expression of all five genes were upregulated in the midgut but the larval development was delayed. Biochemical assays were consistent with the increased protease activity exhibited by SPs in the midgut after treatment with β-Cypermethrin. Taken together, these findings provide evidence for the hypothesis that enhanced SP activity may play an indirect role in relieving the toxicity stress of insecticide in *B. dorsalis*.

## 1. Introduction

The oriental fruit fly, *Bactrocera dorsalis* (Hendel), is one of the most destructive pests in the world and causes huge financial losses worldwide [1]. Damages caused by the oriental fruit fly include both punctures into the fruits and vegetables during oviposition and feeding on the fruit pulp by the developing larvae [2]. Since the biological and ecological traits of *B. dorsalis* include invasive ability (high mobility, dispersive powers, and fecundity), wild distribution, and economic significance, it is ranked high on the quarantine target lists [3].

The insect digestive proteases catalyze the dietary protein to release the free amino acids and supply essential nutrients for growth and development. All insect digestive proteases can be classified into three classes: aspartic (also called carboxyl or acid), cysteine (also called thiol), and serine proteases [4]. Among them, serine proteases (SPs) are considered to be important proteolytic enzymes. Most insects were presumed to use SPs with trypsin or chymotrypsin-like specificities to digest their dietary protein [5]. It is known that SPs represent the major proteolytic enzymes in the midgut of many lepidopterans and dipterans [6]. In some lepidopteran insects, SPs are known to dominate in the larval gut environment and contribute to about 95% of the total digestive activity [7]. They are also dominant proteolytic enzymes in the midgut of *Drosophila* [8].

SPs are a diverse group of proteolytic enzymes. Almost one-third of all proteases can be classified as SPs, named for the nucleophilic Ser residue at the active site. SPs clan of endoproteolytic enzymes can be further divided into families and subfamilies. The S1.A subfamily includes trypsin, chymotrypsins, elastases, and some recently identified serine collagenases [9], which are involved in a number of critical physiological processes, such as digestion, hemostasis, apoptosis, signal transduction, reproduction, and the immune response [10].

Due to the high effectiveness, low toxicity to non-target organisms, and easy biodegradability, pyrethroids are used more preferably over organochlorines and organophosphates [11]. Most pyrethroids contain cyclopropane carboxylic acid (or a moiety equivalent group) linked to aromatic alcohols through a central ester (or ether) bond. Modifications to this basic structure are designed to increase insecticidal potency or photostability [12]. They act on the insect nervous system by slowing action potential decay, and thereby initiating repetitive discharges in motor and sensory axons. Electrophysiological studies suggested that these phenomena result from modification of gating kinetic of neuronal, voltage-sensitive sodium channels [13]. In insects, the metabolic enzymes that are implicated in the detoxification of pyrethroids include cytochrome P450 monooxygenases (P450s), esterases, and glutathione *S*-transferases (GSTs) [14]. Our previous studies found that the overexpression of some GSTs and P450 genes in *B. dorsalis* following exposure to the widely used pyrethroid, β-Cypermethrin [15–17], indicating that both GSTs and P450s play important roles in the stress response of *B. dorsalis* to β-Cypermethrin. In addition, the target enzyme such as acetylcholinesterase (AChE) upon the exposure to β-Cypermethrin in *B. dorsalis* was also well documented [18]. However, the potential insecticide modulation of digestive and energy metabolic enzyme has been an object of little attention. Previous studies have found that in insects the digestive and metabolic energy enzymes play important roles in mitigating fitness cost associated with insecticide resistance [19–22], and it has been confirmed that some xenobiotics could modulate the activity of not only the detoxification enzymes, but also modify the energy and digestive enzymes in an unspecified manner [23,24]. Detoxification is an energy consuming process, it is plausible that the energy utilized originally for normal development and reproduction may be disturbed and channeled toward detoxification. Therefore, we hypothesized that exposure to insecticides may enhance protein digestion in insects resulting in additional energy, and increased availability of amino acids for protein synthesis. This in turn will supply the materials to produce the detoxification apparatus without costing energy for other physiological processes. In this study, we identified five SP genes in the midgut of *B. dorsalis* and determined their development–specific expressions and transcriptional profiles in larvae upon exposure to β-Cypermethrin. In addition, the activities of SPs in the midgut of *B. dorsalis* larvae were also measured. The results might provide some evidence for the indirect role of SPs in insecticide tolerance.

## 2. Results

### 2.1. β-Cypermethrin Intake Slows Larval Development

**Figure 1.**
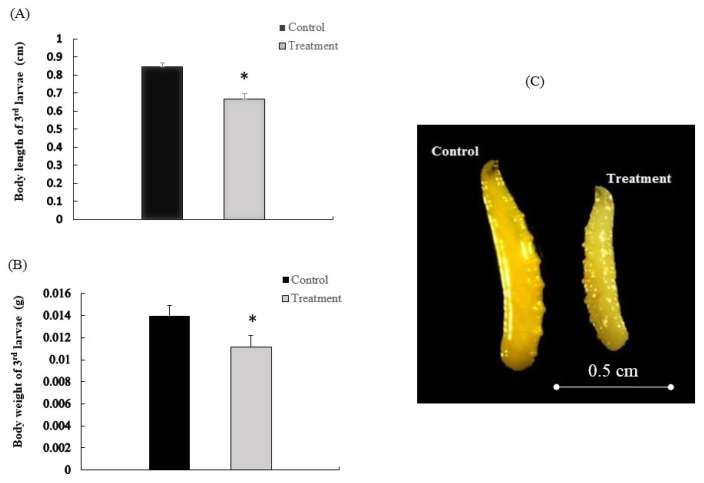
Body length (**A**), weight (**B**) and morphological characteristics (**C**) of 3rd instar larvae treated with (treatment) or without (control) β-Cypermethrin. * indicates significant difference between treatment and control (Independent sample *t*-test, *p* < 0.05).

Feeding β-Cypermethrin containing diet decreased the average length of 3rd instar larvae from 0.85 ± 0.028 to 0.67 ± 0.021 cm (Figure 1A). Similarly, β-Cypermethrin intake also decreased the average larval body weight from 13.98 ± 0.91 to 11.12 ± 1.09 mg (Figure 1B). In addition, severe effects on overall larval morphology were observed when the insects were fed with β-Cypermethrin containing diets (Figure 1C).

### 2.2. Effect of β-Cypermethrin on Protease Activities

Activities of SPs were determined using BApNA and L-TAME as substrates for amidolytic and esterolytic activities, respectively. The results showed that in both processes, SP activities in the treatment group were significantly higher than those in the control group. For BApNA hydrolysis, the specific activity of SPs from the treatment group was 450.00 ± 32.97 nmol/mL/min/mg, which was significantly higher than that in the control group (302.67 ± 79.93 nmol/mL/min/mg) (*p <* 0.05; Table 1). Similarly, for the L-TAME hydrolysis, the specific activity of SPs from the treatment group was 266.00 ± 13.89 nmol/mL/min/mg, which was significantly higher than that in the control group (201.33 ± 16.92 nmol/mL/min/mg) (*p <* 0.05; Table 1).

### 2.3. Amino Acid Similarities

The full-length cDNA sequences of all five SPs consisted of 744–798 nucleotides that encoded proteins with 247–265 amino acid residues and were named as *BdSP1*, *BdSP2*, *BdSP3*, *BdSP4*, and *BdSP5*, respectively. These sequences were deposited in GenBank and the relative accession numbers were: GAAP01000017, GAAP01000019, GAAP01000020, GAAP01000021, and GAAP01000022.

Protein sequence alignments of BdSPs with other known insect SPs revealed the presence of the hallmark SP feature, which is the catalytic triad consisting of His57, Asp102, and Ser195 (chymotrypsin numbering) with in the highly conserved sequences of TAAHC, DIAL, and GDSGGP, respectively, in most SPs (Table 2). These motifs were easily identified in all the predicted amino acid sequences.

**Figure 2.**
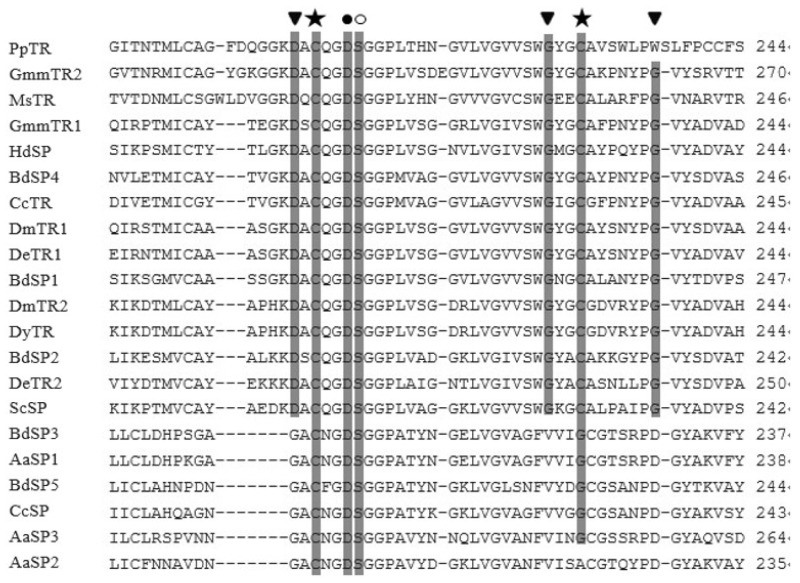
Protein alignment of insect SPs. Protein alignment of all BdSPs used in this analysis from positions 180–240. Residues of importance are represented as follows: (▼) Ser195 catalytic triad residues, (★) accessory catalytic residues, (●) the third and final disulfide bridge and (○) Asp194 where position 1 (Ile/Val) is buried after activation of the mature peptide.

During the analysis we also found that 14 among 21 SP sequences including *BdSP1*, *BdSP2* and *BdSP4*, and other known insect trypsins contained Asp190, Gly216, and Gly226, while the remaining sequences (*BdSP3* and *BdSP5*) contained Gly, Val and Asp at positions 190, 216, 226, respectively (Figure 2).

### 2.4. Phylogenetic Analyses

**Figure 3.**
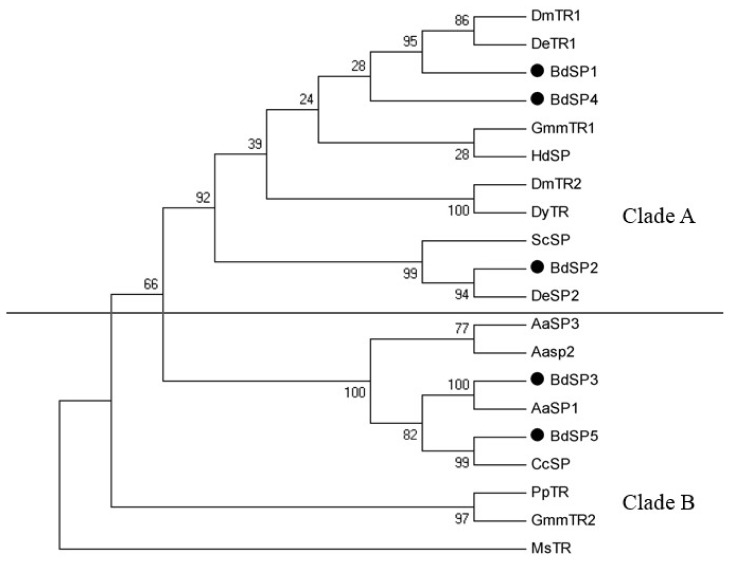
The maximum likelihood phylogenetic tree of SPs. The amino acid segments aligned in Figure 3 were used in this analysis. Clade A corresponds with the known trypsin and *BdSP1*, *BdTSP2* and *BdSP4*, clade B represents other SPs along with *BdSP3* and *BdSP5*.

To analyze the sequence homology and phylogenetic relationships, sixteen other insect SPs were downloaded from GenBank and aligned with the five *BdSP* gene sequences (Table 2). The phylogenetic tree constructed using the maximum likelihood method had two major clades (Figure 3) with all known trypsins along with *BdSP1*, *BdSP2*, and *BdSP4* clustering on the same clade. *BdSP1* was most closely related to *DmTR1* and *DeTR1* from *Drosophila melanogaster* and *D. erecta,* respectively. *BdSP4* was in clade A and shared a common branch with *BdSP1.* Similarly, *BdSP3*, *BdSP5* and other SPs sequences were clustered together in clade B indicting a high sequence similarity. In clade B, *BdSP3* and *BdSP5* were clustered with *AaSP1* from *Aedes aegypti* and *CcSP* from *Ceratitis capitata,* respectively, with 100% bootstrap. Overall the topologies and distances resolved by the maximum likelihood analyses were also supported by the distance-neighbor-joining analyses (data not shown).

### 2.5. Developmental Expression Profiles of BdSPs

qRT-PCR results revealed that all five SPs were highly expressed in the larvae, followed by the adults. The presence of *BdSPs* in both eggs and pupae were also detected, but at lower levels. Among the five genes, the expression level of *BdSP2* was the highest in larvae and adult (Table 3).

### 2.6. Temporal Expression of BdSPs in Response to β-Cypermethrin

**Figure 4.**
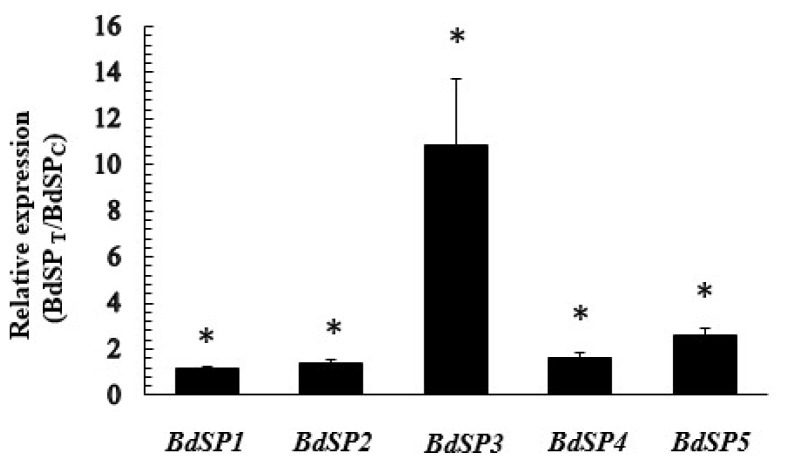
Transcription profiles of the five different *B. dorsalis* SPs (*BdSPs*) in larvae exposed to β-Cypermethrin. Relative expression was calculated as $\frac{2^{\left[\text{CT}({BdSP}_{C})-\text{CT}({BdSP}_{T})\right]}}{2^{\left[\text{CT}({EF}_{C})-\text{CT}({EF}_{T})\right]}}$. *BdSP_T_* and *BdSP_C_* represent target genes from the treatment and control groups, respectively. *EF* represents reference gene (*EF1α*). * indicates significant difference in expression level of target genes between control and treatment (Independent sample *t*-test, *p* < 0.05).

The transcriptional changes of all five *SP* genes transcripts in larvae exposed to β-Cypermethrin determined by qRT-PCR which showed significant upregulation of *BdSP3, BdSP5, BdSP1, BdSP2*, and *BdSP4* in larvae following the treatment with β-Cypermethrin, especially for *BdSP3*. When compared to the control, all five genes registered 10.9-, 2.6-, 1.2-, 1.4- and 1.6-fold increase, respectively (Figure 4). Statistical analyses showed that among the genes, the expression of two genes (*BdSP3* and *BdSP5*) were significantly upregulated compared to the control (*p* < 0.05).

## 3. Discussion

SPs are dominant proteolytic enzymes in the insect midgut [8] and can be distinguished by the presence of the Asp-His-Ser “charge relay” system or “catalytic triad” [9]. They are present in all phylogenetic kingdoms including viruses and are involved in many physiological processes [10]. Particularly, the trypsins and chymotrypsins play important roles in insect food digestion, immune defense, and zymogen activation [26]. In addition, SPs and SP homologs constitute the second largest family of genes in the *D. melanogaster* genome [27]. To date, the genes encoding trypsins and chymotrypsins have been cloned and characterized in several insect species [27–29]. This study is the first report on biochemical and molecular characterization of SPs in *B. dorsalis*.

It is well recognized that toxic substances may induce many biochemical changes preceding cellular and systemic dysfunction in animals [30]. In this study, we found the overexpression of SPs and the enhanced SPs activity in *B. dorsalis* upon the exposure of β-Cypermethrin, which may probably be explained in this way: the detoxification process is known to draw energy allocated to normal development and therefore, absence of energy supplement may impair the normal development and reproduction in insects [31,32]. To relieve the effect of insecticide, when treated with β-Cypermethrin insects might enhance protein digestion. The enhanced hydrolytic enzyme activity will supply additional energy and amino acids for protein synthesis. This will supply the materials to produce the detoxification apparatus for reducing the cost of energy originally utilized by normal physiological processes.

The similar complementary mechanisms have also been observed in some other insects confronted under environmental stress. It has been reported that there was an increase in carbohydrate metabolism induced by organophosphorus insecticide in silkworm. A significant decrease in pyruvate levels and increase in lactate levels were observed in the hemolymph and fat body of silkworm, suggesting the utilization of pyruvate through the tricarboxylic acid (TCA) cycle to meet the required energy demands to counteract the insecticide toxicity [24]. In a similar study, the sharp elevation in protease activity and free amino acid levels were also observed in the hemolymph and fat body of silkworms when exposed to lethal and sublethal doses of fenitrothion and ethion [33]. Besides, it was found that the enhanced trypsins and cysteine protease activities play important roles in mitigating physiological costs associated with the maintenance of insecticide resistance in studies on maize weevil [19,20]. Taken together, it is reasonable to assume that the higher SP activities in *B. dorsalis* might play an indirect role in mitigating the stress caused by β-Cypermethrin.

We obtained the full-length cDNA sequences of five SPs on the base of *B. dorsalis* midgut transcriptome [34]. All the five SPs contained six conserved cysteine residues that correspond to the three disulfide bonds characteristic of arthropod serine proteases [35]. They also had the catalytic triad “Asp-His-Ser”, indicating that these putative proteins were indeed SPs. To predict the substrate specificity of the SPs, we identified the primary substrate–binding pocket (residues 190, 216 and 226 in chymotrypsin) in the aligned sequences [25]. SPs that contain Asp190, Gly216, and Gly/Ala/Ser226 are predicted to have trypsin-like specificity. Therefore we concluded that *BdSP1*, *BdSP2* and *BdSP4* are trypsin-like SPs. (Table 4). Although the result of sequence analysis showed that *BdSP3* (69%) may be trypsin-like SPs, and *BdSP5* (41%) may belong to chymotrypsin-like SPs, our protein alignment did not reveal the hallmark of trypsin (Asp190, Gly216 or Gly/Ala/Ser226) or chymotrypsin (Ser189, Gly216, and Gly226); rather Gly, Val and Asp, were replaced in these positions, respectively. Therefore, we could not unequivocally conclude that all *BdSPs* are trypsin-like SPs. The final classification of *BdSP3* and *BdSP5* is required for further clarification.

The determination of SPs’ activities provided some evidences for our hypothesis, but we also determined the expression profiles of five SPs genes after exposure to β-Cypermethrin by qRT-PCR. Consistent with the enhanced SP activities, we observed that there was a significant increase in expression levels of SP transcripts. Although the increase was not dramatic, it still suggested the trend of upregulation of *SPs* genes, and probably there are other SP genes playing important roles in the process. It was the desirable result that there was no significant difference in larval morphology between control and treatment, which would be more in favor of our hypothesis. However, in fact, after the treatment of β-Cypermethrin, the development of *B. dorsalis* larvae was delayed. Maybe it can be explained in this way that the stress responses of insects are complicated processes, and the internal changes we found might be not great enough to directly reflect on larval morphology and completely cover the toxicity stress caused by β-Cypermethrin. The five genes were highly expressed in the midgut of larvae suggesting a high energy requirement at this stage, which is consistent with the developmental characteristics of *B. dorsalis* larvae that feed actively on host fruits and vegetables. The accumulation of nutrition and energy at the larval stage guarantees normal development and reproduction in *B. dorsalis*. After larval hatching, the enzymes associated with digestion and absorption was highly expressed to satisfy the strong demand for nutrition and energy of *B. dorsalis* larvae. This is also the reason why we chose the larval stage of *B. dorsalis* in our study. As expected, the upregulation of these five SPs genes and the increase of serine-protease activity after exposure to β-Cypermethrin demonstrate that SPs do indirectly mitigate the toxicity stress of β-Cypermethrin in *B. dorsalis*.

## 4. Experimental Section

### 4.1. Insects

The laboratory stock of *B. dorsalis*, originally collected in 2008 from Hainan province, China, was cultured in the insectary at 27 ± 1 °C, 70% ± 5% relative humidity, 14 h light: 10 h dark photoperiod. Larvae were reared on an artificial diet described previously [34].

### 4.2. Exposure to β-Cypermethrin

About fifty newly emerged *B. dorsalis* larvae were reared on an artificial diet containing 0.33 μg/g β-Cypermethrin or the control diet with acetone (solvent used to dissolve β-Cypermethrin) from 1st to 3rd instar, which took about five days. At the end of 3rd instar, body length and mass of the surviving larvae were weighed in both control and treated groups. All experimental groups were maintained under the same conditions (27 ± 1 °C, 70% ± 5% relative humidity, and 14 h:10 h (L:D) photoperiod) in culture dishes. The choice of β-Cypermethrin concentration (0.33 μg/g in artificial diet) was based on our previous study [36]. The β-Cypermethrin (95%) was provided by Institute for Control of Agrochemicals of Sichuan Province, Chengdu, China.

### 4.3. Preparation of Enzyme Extracts

Enzyme extracts were prepared from the midguts of larvae by dissecting on ice under a stereomicroscope (Olympus SZX12, Tokyo, Japan), Treatment and control group both contained three biological replications, and 30 larvae midguts were collected in each sample. The dissected midguts were frozen in liquid nitrogen, homogenized in 0.1 M Tris-HCl (pH 8.2 containing 10 mM (CaCl_2_) and 2 mL of the homogenate or crude extract was centrifuged at 12 000*g* for 5 min at 4 °C and filtered through a 0.25 μm filter membrane. The supernatant was centrifuged again at 17,500*g* for 25 min at 4 °C, and 2 mL of the final supernatant was collected and used as the enzyme source.

### 4.4. Protein Concentration and Protease Activity

Protein content in the enzyme extracts was determined based on described methods using bovine serum albumin as the standard [37]. Activities of SPs were determined using *N*-α-benzoyl-dl-arginine *p*-nitroanilide (BApNA) and *N*-α-*p*-tosyl-l-Arg methyl ester (l-TAME) as substrates for amidolytic and esterolytic activities, respectively. Amidolytic activity was measured with BApNA as substrate [32], at a final concentration of 1.7 mM in glycine sodium-hydroxide buffer (pH 10.5) containing 7.4% dimethylformamidine (DMF, *v*/*v*) at 37 °C [38,39]. Esterolytic activity was measured with l-TAME; 1 mM as substrate in 0.1 M Tris-HCl buffer (pH 8.2) at 25 °C [40]. Each assay in the control and treated groups was repeated three times.

### 4.5. RNA Isolation and First-Strand cDNA Synthesis

Total RNA was extracted from the midgut of control and treated larvae using an RNAeasy Micro Kit (Qiagen, Hilden, Germany). Total RNAs from whole bodies of insects were isolated at each developmental stage (eggs, larvae, pupae, and adults) using TRIzol reagent (Biomed, Beijing, China). About 30 individuals were dissected from each of the control and treated groups, and each developmental stage. In addition, three biological replicates were maintained per sample.

The midguts were dissected under a stereomicroscope (Olympus SZX12, Tokyo, Japan) and placed in a 1.5-mL centrifuge tube containing the RNA store reagent (Tiangen, Beijing, China). The collected midguts and insect bodies from different life stages were homogenized immediately in liquid nitrogen. RNA was quantified by measuring the absorbance at 260 nm using a NanoVue UV-Vis spectrophotometer (GE Healthcare Bio-Science, Uppsala, Sweden). The purity of all RNA samples was assessed based on absorbance ratio at OD260/280 and OD260/230, and the integrity of RNA was checked on a 1% agarose gel. The RNA extraction using the RNAeasy Micro Kit included an on-column genomic DNA elimination step that efficiently removed genomic DNA from the total RNA preparations. The total RNA extracted by TRIzol reagent was treated separately by DNase I (Promega, Madison, WI, USA). First strand cDNA was synthesized using a PrimeScript® RT reagent Kit (Takara, Dalian, China).

### 4.6. Sequence Analysis and Phylogenetic Tree Construction

Five SP gene sequences were obtained from the midgut transcripts of *B. dorsalis* larvae [34]. DNA sequences were edited with DNAMAN 5.2.2 (Lynnon Pointe-Claire, Quebec, Canada) and the deduced amino acid sequences of all five SPs genes were trimmed and aligned using Clustal X [41]. Homologous SP protein sequences were searched by BLAST in the NCBI database (http://blast.ncbi.nlm.nih.gov/Blast.cgi). Phylogenetic tree was constructed with MEGA 5.0 using the Maximum likelihood method. Bootstrap values were calculated with 1000 replications. The *MsTR* of *Manduca sexta* was used as outgroup in constructing phylogenetic tree.

### 4.7. Analysis of SP Gene Expression

We analyzed the expression patterns of all five SP genes in each developmental stage (eggs, larvae, pupae, and adults) of *B. dorsalis* and also their expression in both the control and treatment group by quantitative real-time-PCR (qRT-PCR). Based on standard protocols, *α-tubulin* and *EF1α* were selected as reference genes for the analyses [42].

qRT-PCR was conducted on a Stratagene Mx3000P System in a total reaction volume of 20 μL containing 500 ng cDNA, 10 μL GoTaq® qPCR Master Mix (Promega, Madison, WI, USA), 10 pmol of each primer (Table 4), and double distilled water. All reactions were performed under the following conditions: 95 °C for 30 s, 40 cycles of 95 °C for 15 s, 60 °C for 30 s, and 72 °C for 30 s. To verify the specificity of the amplicon for each primer pair, a dissociation curve was included from 60–95°C at the end of each qRT-PCR run. A cDNA dilution series (1, 1/3, 1/9, 1/27, and 1/81) with the sample cDNA was used to construct the standard curve and calculate the efficiency of amplification. The PCR efficiencies of each SP gene and the references were calculated using Mxpro-Mx3000P version 3.20 (Agilent-Stratagene, Santa Clara, CA, USA). Relative expression levels were calculated by the comparative CT method [43].

### 4.8. Statistical Analysis

Differences in gene expression levels among the developmental stages were analyzed by one-way analysis of variance (ANOVA), followed by Duncan’s multiple range tests in SPSS 17.0 (SPSS Armonk, New York, NY, USA). All other data were analyzed with independent sample *t*-test for significance (*p* < 0.05) with SPSS 17.0.

## 5. Conclusions

In summary, the increases of *SP* gene expression and enzyme activity after exposure to β-Cypermethrin may reflect the potential role of SPs in relieving the toxicity stress of β-Cypermethrin. Additional studies of SP genes will help elucidate their possible role in different life stages at the molecular level.

## Figures and Tables

**Table 1.** Specific activity of SPs in the midgut of 3rd instar larva of *B. dorsalis*.

Groups	Specific activity (nmol/mL/min/mg)	

	BApNA	L-TAME
Control	302.67 ± 79.93	201.33 ± 16.92
Treatment	450.00 ± 32.97 *	266.00 ± 13.89 *

Each value represents the mean (M ± SE) of three replications.

* represents significant difference (Independent sample *t*-test, *p* < 0.05).

**Table 2.** List of serine proteases used in the alignment and phylogenetic analyses.

Species	Serine protease	GenBank Accession number	Conserved regions a			Length	Enzyme specificity b

			TAAHC	DIAL	GDSGGP		
*Bactrocera. dorsalis*	BdSP1	GAAP01000017	SATHC	DIGL		265	T(DGG)
*Bactrocera. dorsalis*	BdSP2	GAAP01000019		DIAI		259	T(DGG)
*Bactrocera. dorsalis*	BdSP3	GAAP01000020		DLAL		247	E(GVD)
*Bactrocera. dorsalis*	BdSP4	GAAP01000021				258	T(DGG)
*Bactrocera. dorsalis*	BdSP5	GAAP01000022				257	E(GVD)
*Glossina morsitans morsitans*	GmmTR1	ACB98719.1		DVAV		256	T(DGG)
*Glossina morsitans morsitans*	GmmTR2	ADD19605.1		DYSL		281	T(DGG)
*Aedes aegypti*	AaSP1	XP_004520098.1		DLAL		248	E(GVD)
*Aedes aegypti*	AaSP2	XP_001659492.1				247	E(GVD)
*Aedes aegypti*	AaSP3	XP_001659851.1		DIAV		274	E(GVD)
*Ceratitis capitata*	CcSP	XP_004520096.1		DVAL		254	E(GVD)
*Ceratitis capitata*	CcTR	XP_004517776.1		DIAV		257	T(DGG)
*Drosophila melanogaster*	DmTR1	AAA17449.1		DIVI		253	T(DGG)
*Drosophila melanogaster*	DmTR2	NP_525112.1		DIAI		256	T(DGG)
*Drosophila erecta*	DeTR1	XP_001976081.1		DIAV		256	T(DGG)
*Drosophila erecta*	DeTR2	XP_001976082.1		DVGI		262	T(DGG)
*Drosophila yakuba*	DyTR	XP_002091228.1		DIAI		256	T(DGG)
*Phlebotomus papatasi*	PpTR	AAM96943.1		DFSL		268	T(DGW)
*Manduca sexta*	MsTR	P35046.1		DIAI		256	T(DGG)
*Hypoderma diana*	HdSP	ACF98290.1		DVAI		256	T(DGG)
*Stomoxys calcitrans*	SsSP	AAC39131.1		DVAV		254	T(DGG)

a If not listed, sequences are identical to the conserved TAAHC, DIAL, or GDSGGP;

b enzyme specificity predicted based on Perona and Craik (1995) [25] (SP, Serine Protease; TR, trypsin).

**Table 3.** Expression patterns of five *BdSPs* in different life stages of *B. dorsalis*.

Genes	Relative expression			
	
	Egg	Larva	Pupa	Adult
*BdSP1*	1.0	7.48 ± 0.93	0.05 ± 0.02	1.61 ± 0.51
*BdSP2*	1.0	189.62 ± 23.11	0.47 ± 0.16	118 ± 28.69
*BdSP3*	1.0	4.04 ± 0.86	0.45 ± 0.22	2.21 ± 0.03
*BdSP4*	1.0	6.71 ± 1.72	0.67 ± 0.25	1.10 ± 0.19
*BdSP5*	1.0	7.86 ± 1.48	0.04 ± 0.02	3.56 ± 0.10

**Table 4.** Primers used for qRT-PCR.

Genes	GenBank accession number	Primer name and sequence (5′-3′)	PCR efficiency (%)	*R*^2^
*BdSP1*	GAAP01000017	S: ACACACTCGGGTTTTAGCGT A: GAGGCGCAATCTTCACGTTG	106.25	0.981
*BdSP2*	GAAP01000019	S: AGAGGACGTATTGTTGGCGG A: CGGCACGGATTCTCAAAACC	96.17	0.993
*BdSP3*	GAAP01000020	S: ATCCCACGGGTCGTGTAGTA A: GTTGTGCCTGTTTCGACCAC	108.43	0.986
*BdSP4*	GAAP01000021	S: CAACGTGAAGATTGCGCCTC A: CCTTTTTGGCACAGCCTTCC	91.95	0.989
*BdSP5*	GAAP01000022	S: TTCCCCCATCAGGTCTCAC A: CCGAGTGTGCGTTGGATAC	94.61	0.988
